# Effect of Small Reaction Locus in Free-Radical Polymerization: Conventional and Reversible-Deactivation Radical Polymerization †

**DOI:** 10.3390/polym8040155

**Published:** 2016-04-20

**Authors:** Hidetaka Tobita

**Affiliations:** Department of Materials Science and Engineering, University of Fukui, 3-9-1 Bunkyo, Fukui 910-8507, Japan; tobita@matse.u-fukui.ac.jp; Tel.: +81-776-27-8775

**Keywords:** emulsion polymerization, radical polymerization, polymerization rate, theory, reversible-addition-fragmentation chain-transfer (RAFT), stable-radical-mediated polymerization (SRMP), atom-transfer radical polymerization (ATRP)

## Abstract

When the size of a polymerization locus is smaller than a few hundred nanometers, such as in miniemulsion polymerization, each locus may contain no more than one key-component molecule, and the concentration may become much larger than the corresponding bulk polymerization, leading to a significantly different rate of polymerization. By focusing attention on the component having the lowest concentration within the species involved in the polymerization rate expression, a simple formula can predict the particle diameter below which the polymerization rate changes significantly from the bulk polymerization. The key component in the conventional free-radical polymerization is the active radical and the polymerization rate becomes larger than the corresponding bulk polymerization when the particle size is smaller than the predicted diameter. The key component in reversible-addition-fragmentation chain-transfer (RAFT) polymerization is the intermediate species, and it can be used to predict the particle diameter below which the polymerization rate starts to increase. On the other hand, the key component is the trapping agent in stable-radical-mediated polymerization (SRMP) and atom-transfer radical polymerization (ATRP), and the polymerization rate decreases as the particle size becomes smaller than the predicted diameter.

## 1. Introduction

The rate of polymerization, *R*_p_ in free-radical polymerization is represented by:

(1) $$R_{p}=k_{p}[M][R^{•}]$$

where *k*_p_ is the propagation rate constant, [M] is the monomer concentration, and [R^•^] is the active radical concentration.

At the same monomer concentration, the polymerization rate is higher for larger radical concentration, [R^•^]. For usual bulk polymerization, [R^•^] is determined from the balance of initiation rate *R*_I_ and termination rate *R*_t_ under stready state, with *R*_I_ = *R*_t_ = *k*_t_[R^•^]^2^, leading to obtain:

(2) $$[R^{•}]=\sqrt{\frac{R_{I}}{k_{t}}}$$

where *k*_t_ is the termination rate constant. Note that the convention of termination rate, *R*_t_ = *k*_t_[R^•^]^2^ that does not involve the coefficient 2 is used ([1], p. 12).

**Figure 1:**
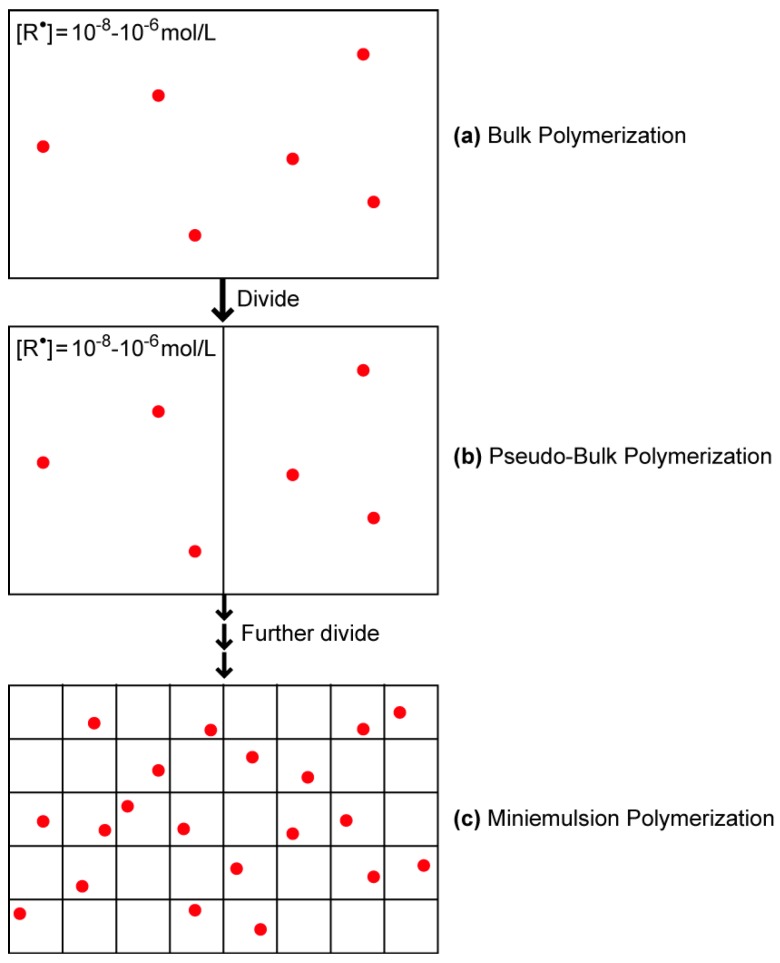
Schematic representation of the radical concentration in (**a**) bulk; (**b**) pseudo-bulk; and (**c**) miniemulsion polymerization.

The bimolecular termination rate in free-radical polymerization is very fast, and Equation (2) leads to extremely small radical concentration [R^•^] in the order of 10^−8^ to 10^−6^ mol/L in usual free-radical polymerization, as schematically represented by (a) bulk polymerization in Figure 1, where red dots are the active radicals.

In general, concentration does not change even when the reaction system is divided, as shown in Figure 1b; pseudo-bulk polymerization. On the other hand, when the polymerization locus is further divided into smaller particles with a few hundred nanometers, the polymerization behavior may change significantly. Each square section in Figure 1c represents a polymer particle in miniemulsion polymerization. The radicals located in other particles cannot terminate each other, and therefore, when the radical generated in the water phase enters a particle without a radical, it may stay to propagate in that particle and the number of radical in it is unity. On the other hand, if the radical enters a particle already having a radical, because the radicals that exist in the same particle possess a very high concentration, they terminate each other instantaneously. Each particle contains zero or one radical, and if the radicals do not exit from the particles, the average number of radicals in a particle is 0.5. This kind of radical isolation effect is sometimes referred to as the compartmentalization ([2], p. 65). The radical concentration in the reaction locus can become much larger than the corresponding bulk polymerization, leading to a much larger rate of polymerization. The average radical concentration in a particle, [R^•^]_p_ is represented by:

(3) $${\left[R^{•}\right]}_{p}=\frac{\bar{n}}{N_{A}v_{p}}=\frac{6\bar{n}}{\pi N_{A}d_{p}{}^{3}}$$

where $\bar{n}$ is the average number of radicals in a particle, $N_{A}$ is the Avogadro constant, $v_{p}$ is the particle volume, and *d*_p_ is the particle diameter.

Table 1 shows the calculated concentration of a single molecule in a particle with various values of diameter. When the particle diameter is as large as *d*_p_ = 1000 nm, the concentration of a single molecule is negligibly small, compared with [R^•^]_bulk_, which is in the order of 10^−8^ to 10^−6^ mol/L. The particle with *d*_p_ = 1000 nm may contain a large number of radicals in it, and the polymerization behavior is essentially the same as in bulk polymerization. This is the case for suspension polymerization. In general, when the average number of radicals in a particle is larger than 2, the pseudo-bulk polymerization kinetics can be applied [3], at least approximately.

On the other hand, when the particle diameter is smaller than *ca.* 100 nm, even with a single radical in a particle, the concentration may be much larger than usual bulk polymerization, leading to a larger polymerization rate than the corresponding bulk polymerization.

In this article, simple formulas to quantitatively predict the particle diameter below which the polymerization rate becomes much different from the bulk polymerization are elucidated, based on the high single-molecule concentration in a small particle. I already discussed the threshold diameters in the earlier articles [4,5]. However, the statistical variation effect of the key component concentration among the particles was considered at the same time in these articles, which seems to make the discussion rather complicated. In this article, the effect of high single-molecule concentration on the polymerization rate is reorganized, starting from the conventional free-radical polymerization. I mainly refer to the articles of my research group throughout the discussion. This is not because I do not appreciate important contributions by the other research groups deeply, but solely for the theoretical consistency to enhance readability. Readers may find the other interesting aspects of the related topic in refs [2,6,7,8,9,10].

## 2. Conventional Free-Radical Polymerization

To theoretically consider the effect of small reaction locus, it is convenient to envisage an ideal miniemulsion polymerization. With miniemulson polymerization, the initial stage of polymerization that involves nucleation may be complicated [11]. However, after the initial stage, each polymer particle could be considered as an isolated microreactor, to which a radical enters occasionally. In this article, monodisperse particles are assumed to consider the size effect of polymerization locus. The present discussion applies also for the *ab initio* emulsion polymerization when compared with the corresponding bulk polymerization at the same monomer concentration.

Consider the threshold diameter below which the polymerization rate increases significantly by decreasing the particle size in conventional free-radical polymerization. The average radical concentration in the particles, [R^•^]_p_ is given by Equation (3), which shows that the [R^•^]_p_–value increases significantly by decreasing the particle diameter *d*_p_, assuming the value of $\bar{n}$ does not change notably. When [R^•^]_p_ is larger than that in bulk polymerization, the polymerization rate is larger than the bulk polymerization at the same monomer concentration. Therefore, the condition where the miniemulsion polymerization rate is larger than the corresponding bulk polymerization is represented by:

(4) $$\frac{6\bar{n}}{\pi \;N_{A}d_{p}{}^{3}}>{\left[R^{•}\right]}_{\text{bulk}}$$

The radical concentration in bulk polymerization [R^•^]_bulk_ is simply given by Equation (2). The particle diameter below which the polymerization rate becomes larger than in bulk polymerization is given by:

(5) $$d_{{p,R}^{\text{•}}}^{\left(1\right)}={\left(\frac{6\bar{n}}{\pi N_{A}{\left[R^{•}\right]}_{\text{bulk}}}\right)}^{1/3}$$

To represent the threshold diameter, the superscript (1) is used to represent that the effect is caused by a high single-molecule concentration in a small particle, and the subscript R^•^ represents that the key component is the polymer radical.

Consider a simple example to illustrate the theory. Suppose the initiation rate in bulk polymerization is *R*_I_ = 1 × 10^−7^ mol·L^−1^·s^−1^, and the termination rate constant is *k*_t_ = 1 × 10^7^ L·mol^−1^·s^−1^. Assuming a hydrophobic monomer, such as styrene, the average number of radicals for small particles is $\bar{n}$ = 0.5 for a wide range of diameters. In this case, Equation (5) leads to the threshold diameter, $d_{p,R}^{\left(1\right)}$ = 252 nm.

**Figure 2:**
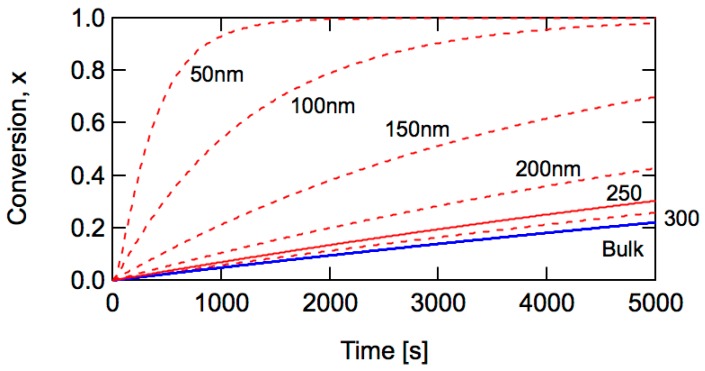
Calculated conversion development for bulk and miniemulsion polymerization with *R*_I_ = 1 × 10^−7^ mol·L^−1^·s^−1^, *k*_t_ = 1 × 10^7^ L·mol^−1^·s^−1^, $\bar{n}$ = 0.5 and *k*_p_ = 500 L·mol^−1^·s^−1^.

Figure 2 shows the calculated results based on the Monte Carlo (MC) simulation method proposed in [3,12], with *k*_p_ = 500 L·mol^−1^·s^−1^. It is clearly shown that the polymerization rate increases significantly for *d*_p_ < 250 nm, which shows excellent agreement with Equation (5).

Note that the initiation occurs in the oil phase in bulk polymerization of hydrophobic monomers, and that occurs in water phase in miniemulsion polymerization. Different initiators need to be used and the initiation rate with respect to the unit volume of oil phase is set to be the same in the MC simulation. The initiation rate in water phase *R*_Iw_ (mol·(L-water)^−1^·s^−1^) satisfies the following relationship.

(6) $$\frac{R_{\text{Iw}}}{N_{T}}=R_{I}v_{p}$$

where *N*_T_ is the total number of particles in unit water phase (L-water)^−1^. The series of miniemulsion polymerizations shown in Figure 2 corresponds to the experiments in which the amount of initiator in the water phase is kept constant and the particle size is changed with a constant monomer/water ratio.

## 3. Reversible-Deactivation Radical Polymerization

### 3.1. Polymerization Rate Expression

In free-radical polymerization, the bimolecular termination reactions of the active radicals are inevitable. Therefore, the living polymerization in which the chain termination reactions are totally absent in a strict sense, is impossible. However, if a large percentage of polymer chains are dormant and can potentially grow further, such free-radical polymerization systems can be regarded as pseudo-living polymerization. By introducing the reversible-deactivation process in free-radical polymerization, polymers having a narrow distribution can be obtained. This type of radical polymerization has been referred to as, “controlled”, “controlled/living”, or “living” radical polymerization. In this article, the IUPAC recommended name [13], reversible-deactivation radical polymerization, RDRP is used.

RDRP belongs to free-radical polymerization, and the polymerization rate expression represented by Equation (1) is still valid. However, to clarify the high single-molecule-concentration effect, it is convenient to use the polymerization rate expression that involves the concentrations of important components and is unique to RDRP.

**Figure 3:**
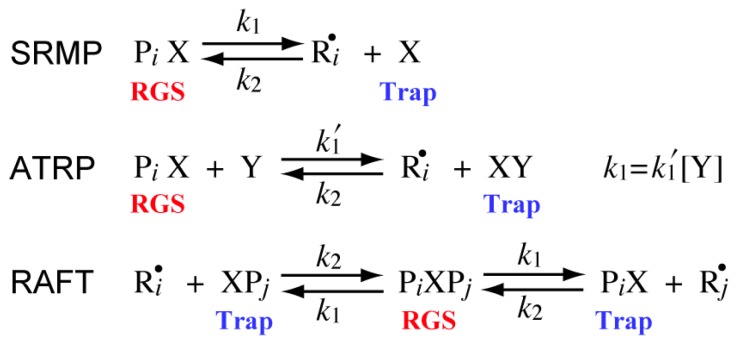
Reversible deactivation reaction scheme in each type of RDRP. In the figure, P*_i_*X or XP*_i_* is the dormant polymer with chain length *i*. $R_{i}^{•}$ is the active polymer radical with chain length *i*.

Figure 3 shows the reversible deactivation reactions in the representative RDRPs, *i.e.*, stable-radical-mediated polymerization (SRMP), atom-transfer radical polymerization (ATRP), and reversible-addition-fragmentation chain-transfer (RAFT) polymerization. In order to formulate the polymerization rate expression for various types of RDRPs in a unified manner, the component that generates an active radical is represented as the radical generating species (RGS), and the component that deactivates an active radical is represented as the trapping agent (Trap) in Figure 3.

Because the lifetime of an active radical in free-radical polymerization is short, normally less than a few seconds, a basic strategy to keep the chain potentially active is to distribute very short active periods throughout the whole reaction time. The rate of deactivation reaction, which is the number of deactivation reactions in a unit volume in a second, is given by:

(7) $$R_{\text{deact}}=k_{2}[\text{Trap}][R^{•}]$$

With respect to a single radical, the frequency of deactivation (s^−1^) is given by:

(8) $$\frac{R_{\text{deact}}}{\left[R^{•}\right]}=k_{2}[\text{Trap}]$$

Therefore, the average time of a single active period, ${\bar{t}}_{\text{act}}$ is given by:

(9) $${\bar{t}}_{\text{act}}=\frac{1}{k_{2}[\text{Trap}]}$$

The magnitude of ${\bar{t}}_{\text{act}}$ is normally in the order of 10^−4^ to 10^−2^ s.

In order to keep a good living condition, the deactivation rate *R*_deact_ must be much larger than the bimolecular termination reaction rate *R*_t_. If not, a large number of dead polymer chains are formed. At the same time, the activation rate *R*_act_ must be much larger than the initiation rate *R*_I_. If not, a large number of new chains are formed, leading to not only broadened molecular weight distribution but also to increased termination frequency.

(10) $$R_{\text{deact}}=k_{2}[\text{Trap}][R^{•}]>>R_{t}$$

(11) $$R_{\text{act}}=k_{1}[\text{RGS}]>>R_{I}$$

For the systems with a very short active period, the polymerization rate is represented by:

(12) $$R_{p}=R_{\text{gen}}{\bar{P}}_{n,\text{SA}}$$

where *R*_gen_ is the radical generation rate (*R*_gen_ = *R*_act_ + *R*_I_), and ${\bar{P}}_{n,\text{SA}}$ is the average number of monomeric units added during a single active period.

Because *R*_act_ >> *R*_I_, the following equation holds.

(13) $$R_{\text{gen}}=R_{\text{act}}=k_{1}[\text{RGS}]$$

The second term in Equation (12), ${\bar{P}}_{n,\text{SA}}$ can be represented by:

(14) $${\bar{P}}_{n,\text{SA}}=R_{\text{add}}{\bar{t}}_{\text{act}}$$

where *R*_add_ is the rate of monomer addition to a single active radical, which is given by:

(15) $$R_{\text{add}}=\frac{k_{p}[M][R^{•}]}{\left[R^{•}\right]}=k_{p}[M]$$

By substituting Equations (9) and (15) into Equation (14), one obtains:

(16) $${\bar{P}}_{n,\text{SA}}=\frac{k_{p}[M]}{k_{2}[\text{Trap}]}$$

From Equations (13) and (16), Equation (12) leads to give the polymerization rate expression unique to RDRP, as follows.

(17) $$R_{p}=k_{p}[M]K\frac{\left[\text{RGS}\right]}{\left[\text{Trap}\right]}$$

where $K=k_{1}/k_{2}$.

Validity of Equation (17) for bulk polymerization under various conditions was examined earlier [4]. The effect of small reaction locus in RDRP is elucidated on the basis of Equation (17).

### 3.2. SRMP and ATRP

In SRMP and ATRP, the position of equilibrium in the reversible reaction shown in Figure 3 is very much toward the RGS side, and [Trap] << [RGS]. The component whose concentration may become larger by the high single-molecule-concentration effect is the trapping agent. Because [Trap] is in the denominator term in Equation (17), the polymerization rate may become smaller than in bulk polymerization, when the particle size is sufficiently small. The condition where the miniemulsion polymerization rate becomes smaller than in bulk is given by:

[Trap]_p_ > [Trap]_bulk_ (18)

where [Trap]_p_ and [Trap]_bulk_ are the trapping agent concentration in the particle and in bulk polymerization, respectively.

The concentration of a single trapping agent in a particle is given by:

(19) $$\left(\text{Single trapping agent concentration in a particle}\right)=\frac{1}{N_{A}\left(\pi \;d_{p}^{3}/6\right)}$$

Therefore, the miniemulsion polymerization rate is expected to be smaller than in bulk polymerization under conditions represented by the following inequality.

(20) $$\frac{6}{\pi \;N_{A}d_{p}^{3}}>{\left[\text{Trap}\right]}_{\text{bulk}}$$

The threshold particle diameter, $d_{p,\text{Trap}}^{\left(1\right)}$, below which the polymerization rate becomes smaller than the corresponding bulk polymerization is given by:

(21) $$d_{p,\text{Trap}}^{\left(1\right)}={\left(\frac{6}{\pi \;N_{A}{\left[\text{Trap}\right]}_{\text{bulk}}}\right)}^{1/3}$$

The trapping agent concentration in bulk polymerization, [Trap]_bulk_ can be determined simply by solving the material balance equation. For SRMP, it can be determined from the following set of differential equations.

(22) $$\frac{d[R^{•}]}{dt}=R_{I}-k_{t}{\left[R^{•}\right]}^{2}+k_{1}[\text{PX}]-k_{2}[R^{•}][X]$$

(23) $$\frac{d[X]}{dt}=k_{1}[\text{PX}]-k_{2}[R^{•}][X]$$

**Figure 4:**
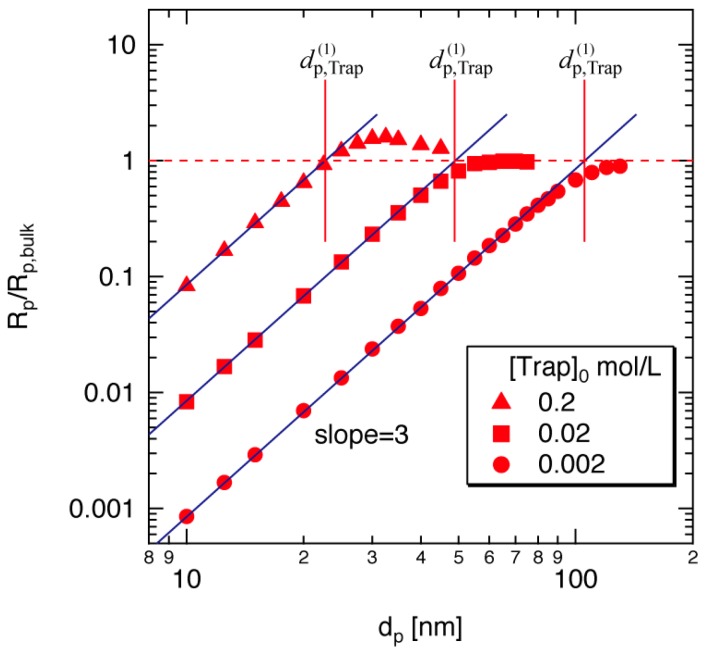
Calculated polymerization rate for the TEMPO-mediated styrene polymerization at 10% conversion with the initial RGS concentration, [RGS]_0_ = 0.2 mol/L. The data (symbols) are taken from [14].

To examine the validity of Equation (21), the calculated results reported by Zetterlund and Okubo [14] are used. The symbols in Figure 4 show the polymerization rate for the given particle size when the conversion is 10%, reported in [14]. The y-axis shows the ratio of the polymerization rate in miniemulsion and the bulk polymerization *R*_p_/*R*_p,bulk_, and the *x*-axis shows the diameter of particles. The initial trapping agent concentration [Trap]_0_ is changed from 0.2 to 0.002 mol/L. For each condition, the threshold diameter $d_{p,\text{Trap}}^{\left(1\right)}$ determined from Equation (21) is shown by the red vertical line. The threshold diameter below which the polymerization rate becomes smaller than that in bulk polymerization agrees reasonably well for every condition, which shows that the simple equation, Equation (21) is convenient to estimate the threshold diameter, without conducting complicated calculations.

Below the threshold diameter, the polymerization rate is proportional to the third power of particle diameter. This is because the single-molecule concentration of [Trap] in a particle, which is in the denominator of Equation (17), is in inverse proportion to the third power of diameter, *d*_p_. Note that [M] and [RGS] inside the particles are large enough to keep the same concentration as the corresponding bulk polymerization.

Figure 4 shows that there exists a particle size region in which the polymerization rate in miniemulsion is slightly larger than in bulk polymerization. This phenomenon results mainly from the statistical variation of the number of trapping agents in a particle [15,16]. In real systems, however, such statistical variation would be blurred by the particle size distribution. In addition, because the degree of acceleration is not very significant, the acceleration region may be difficult to observe experimentally.

In the present theoretical investigation, the exit of trapping agent is not accounted for. If a single trapping agent exits from the polymerizing particle, uncontrolled free-radical polymerization may occur. Because the exit of trapping agent is expected to be more significant for smaller particles, the polymerization rate may not decrease with the third power of *d*_p_. Experimentally, the decrease in polymerization rate by decreasing the particle size is reported [17], but not with *d*_p_^3^.

Smaller polymerization rates in smaller polymer particles make it difficult to conduct the *ab initio* emulsion polymerization in SRMP and ATRP.

### 3.3. RAFT Polymerization

In RAFT polymerization, the concentration of the intermediate, which is RGS in Figure 3, is smaller than that of the trapping agent, and therefore, the high single-molecule concentration effect may be observed for RGS, and [RGS] may become larger than in bulk polymerization. Because [RGS] is the numerator term in the polymerization rate expression given by Equation (17), the polymerization rate may become larger than in bulk, when the particle size is sufficiently small.

**Figure 5:**
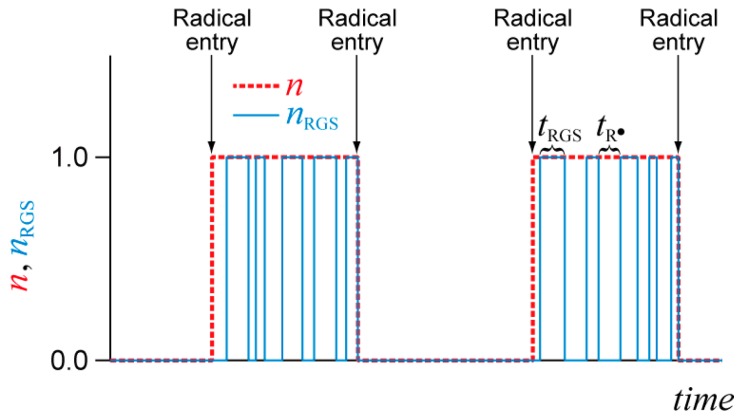
Schematic representation of the zero-one behavior in the conventional free-radical polymerization (**red**) and RAFT (**blue**) miniemulsion polymerization, where *n* is the number of radicals in a particle in the conventional free-radical polymerization and *n*_RGS_ is the number of intermediate molecules in RAFT.

Now, consider the RGS concentration in a particle. Practically, a significant increase in polymerization rate, due to the high-single-molecule concentration effect, occurs with the zero-one behavior in conventional free-radical polymerization. The red dashed line in Figure 5 shows the time change of the number of radicals in a particle for the zero-one system. When the second radical enters the particle, two radicals in the particle terminate each other instantaneously. If the exit of a radical can be neglected, the average number of radicals in a particle is $\bar{n}=0.5$.

When the RAFT agent is introduced, during the growing period in the conventional free-radical polymerization, the number of propagating radicals *n*_R_^•^ and the number of RGS molecules *n*_RGS_ change zero and one alternatively, because of the reversible reaction shown in Figure 3. The blue line shows the number of RGS molecules in a particle. In RAFT, when the second radical enters the particle, both species must be in the active state in order to cause bimolecular termination reaction. The occurrence of the termination reaction could be delayed slightly compared with the conventional free-radical polymerization. However, the monomer consumption during the delayed period could be neglected. In any case, to roughly estimate the threshold diameter what we want to determine is the approximate value of the average number of RGS molecules in a particle, which could be represented by:

(24) $$\left(\text{Average number of RGS molecules in a particle}\right)=\bar{n}\left(1-\phi_{\text{act}}\right)$$

where $\bar{n}$ represents the average number of radicals in a particle when no RAFT agent is used, *i.e.*, in the conventional free-radical miniemulsion polymerization, and $\phi_{\text{act}}$ is the average time fraction of the active period, which is defined explicitly by:

(25) $$\phi_{\text{act}}=\frac{{\bar{t}}_{\text{act}}}{{\bar{t}}_{\text{act}}+{\bar{t}}_{\text{inact}}}$$

In the above equation, the average time of a single active period, ${\bar{t}}_{\text{act}}$ is already given by Equation (9). The average time of a single inactive period, ${\bar{t}}_{\text{inact}}$ can be formulated similarly with what was done for ${\bar{t}}_{\text{act}}$, by considering the frequency, 1/${\bar{t}}_{\text{inact}}$, as follows.

(26) $$\frac{1}{{\bar{t}}_{\text{inact}}}=\frac{R_{\text{act}}}{\left[\text{RGS}\right]}=k_{1}$$

Substituting Equations (9) and (26) into Equation (25), one obtains:

(27) $$\phi_{\text{act}}=\frac{K}{K+[\text{Trap}]}$$

The average concentration of RGS in a small particle can be estimated by:

(28) $${\left[\text{RGS}\right]}_{p}=\frac{\bar{n}\left(1-\phi_{\text{act}}\right)}{N_{A}\left(\pi \;d_{p}^{3}/6\right)}$$

The miniemulsion polymerization rate is expected to become larger than the corresponding bulk polymerization when [RGS]_p_ > [RGS]_bulk_. Equation (28) leads to give the following threshold diameter.

(29) $$d_{p,\text{RGS}}^{\left(1\right)}={\left(\frac{6\bar{n}\left(1-\phi_{\text{act}}\right)}{\pi \;N_{A}{\left[\text{RGS}\right]}_{\text{bulk}}}\right)}^{1/3}$$

#### 3.3.1. Two Conflicting RAFT Models

To determine the RGS concentration in bulk polymerization [RGS]_bulk_, the material balance equations, similarly with Equations (22) and (23), are needed, which depends on the elementary reactions.

It is known that the RAFT polymerization rate shows retardation behavior by increasing the concentration of the RAFT agent. To rationalize the retardation, two conflicting models were proposed. One model [18] assumes that the intermediate species, PXP, which is an inactive radical, terminates with the propagating radical R^•^. This model is called the intermediate termination (IT) model. By representing PXP as RGS, the intermediate termination reaction is represented as follows.

(30) $$\text{RGS}+R^{•}\overset{k_{t,\text{RGS}}}{\to }dead\;polymer\;\left(\text{IT model}\right)$$

where *k*_t,RGS_ is the bimolecular termination rate constant between RGS and the active radical, R^•^.

On the other hand, a slower fragmentation of RGS can also cause retardation [19], which is called the slow fragmentation (SF) model. Both models fit with the bulk polymerization data reasonably well, but the estimated *k*_1_ value for the same reaction system could be more than 10^5^ times larger for the IT model than the SF model [20]. The large difference in *k*_1_ leads to a significant difference in the RGS concentration, *i.e.*, [RGS]_bulk,IT_ << [RGS]_bulk,SF_.

The concentration of RGS in bulk polymerization can be determined from the following set of differential equations.

(31) $$\frac{d[R^{•}]}{dt}=R_{I}+k_{1}[\text{RGS}]-k_{2}[R^{•}][\text{Trap}]-k_{t}{\left[R^{•}\right]}^{2}-k_{t,\text{RGS}}[R^{•}][\text{RGS}]$$

(32) $$\frac{d[\text{RGS}]}{dt}=k_{2}[R^{•}][\text{Trap}]-k_{1}[\text{RGS}]-k_{t,\text{RGS}}[R^{•}][\text{RGS}]$$

(33) $$\frac{d[\text{Trap}]}{dt}=k_{1}[\text{RGS}]-k_{2}[R^{•}][\text{Trap}]$$

With the SF model, *k*_t,RGS_ = 0.

**Figure 6:**
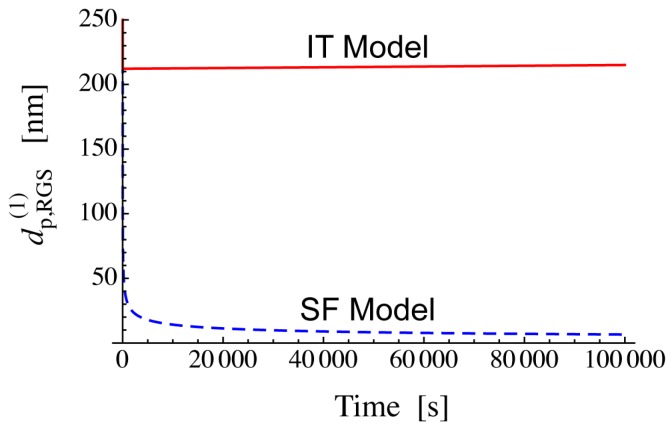
Calculated threshold diameter change during RAFT polymerization. The parameters used are: *R*_I_ = 1 × 10^−7^ mol·L^−1^·s^−1^, *k*_p_ = 500 L·mol^−1^·s^−1^, *k*_t_ = 1 × 10^7^ L·mol^−1^·s^−1^, [M]_0_ = 8 mol·L^−1^ and *k*_2_ = 1 × 10^6^ L·mol^−1^·s^−1^ for both models. For the IT model, *k*_1_ = 1 × 10^4^ s^−1^ and *k*_t,RGS_ = 1×10^7^ L·mol^−1^·s^−1^. For the SF model, *k*_1_ = 0.5 s^−1^ and *k*_t,RGS_ = 0.

The threshold diameter given by Equation (29) shows that a large difference in [RGS]_bulk_ leads to a large difference in $d_{p,\text{RGS}}^{\left(1\right)}$. Figure 6 shows how $d_{p,\text{RGS}}^{\left(1\right)}$ changes during RAFT polymerization, by using a set of representative parameters for dithiobenzoate-mediated styrene polymerization. In the SF model, it takes time to reach the steady state concentration of RGS, and $d_{p,\text{RGS}}^{\left(1\right)}$ decreases slowly during the initial stage of polymerization. The threshold diameter is $d_{p,\text{RGS}}^{\left(1\right)}$= 212 nm for the IT model, while $d_{p,\text{RGS}}^{\left(1\right)}$ is smaller than 10 nm for the SF model.

**Figure 7:**
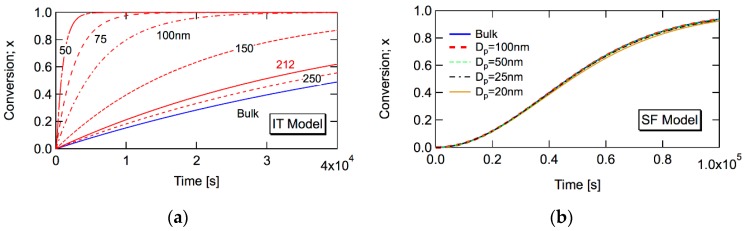
Monte Carlo simulation results for IT and SF model, with the same set of parameters used in Figure 6, based on (**a**) the IT model and (**b**) the SF model. Reproduced from Figure 1 in [23]. © Copyright permission from Wiley-VCH Verlag GmbH & Co. KGaA.

Figure 7a,b show the simulation results for the conversion development during bulk and miniemulsion polymerization, by using the same set of parameters as in Figure 6. For the miniemulsion polymerization, the MC simulation method proposed earlier [21,22] was used. For the bulk polymerization, the following differential equation for the conversion development was used together with Equations (31)–(33).

(30) $$\frac{dx}{dt}=k_{p}(1-x)[R^{•}]$$

In the IT model (Figure 7a), the miniemulsion polymerization shows significant rate increase for *d*_p_ < 212 nm, as predicted by Equation (29). On the other hand, in the case of the SF model (Figure 7b), the $d_{p,\text{RGS}}^{\left(1\right)}$-value is so small (Figure 6), and the polymerization rate is not increased by making the particle size smaller. On the basis of the numerical calculations, using a wide range of parameters, it was concluded that the SF model does not show the polymerization rate increase by decreasing the particle size [23], as long as the given set of parameters cause the retardation behavior in bulk polymerization.

#### 3.3.2. Application of Threshold Diameter to Discriminate RAFT Models

On the basis of a large difference in the threshold diameter between the IT model and the SF model, these two models can be discriminated by the miniemulsion polymerization experiment, *i.e.*, a significant polymerization rate increase by decreasing the droplet size is expected for the IT model, while the SF model does not show the rate increase in miniemulsion polymerization. For the miniemulsion experiments, the polymeric RAFT agent is recommended to use to prevent the exit of RAFT agents from the particles.

**Figure 8:**
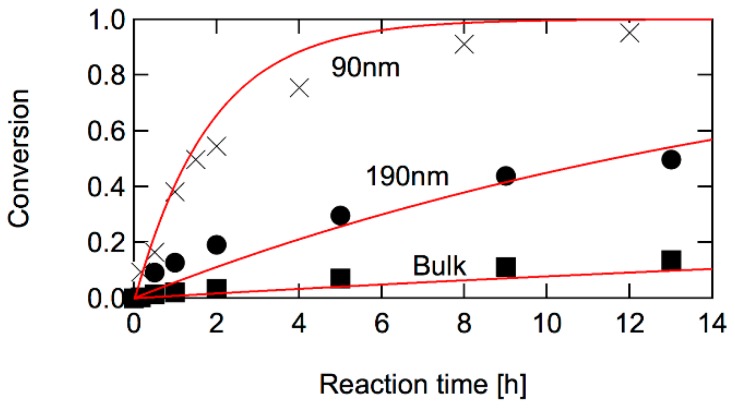
Conversion development during polystyryl dithiobenzoate-mediated styrene polymerization at 60 °C [25]. The solid curves are the calculation results and symbols are the experimental results. For the calculation, the differential equations given by Equations (31)–(34) are solved for the bulk polymerization, and for miniemulsion polymerization, the MC simulation was employed for each fixed diameter. Reproduced from the graphical abstract of [25]. © Copyright permission from Wiley-VCH Verlag GmbH & Co. KGaA.

This model discrimination method was applied for polystyryl dithiobenzoate-mediated styrene polymerization [24,25]. The symbols in Figure 8 show the experimental results. For bulk polymerization, the oil-soluble initiator, AIBN was used, while the water-soluble initiator, potassium persulfate was used in the miniemulsion polymerization experiment. The initiator concentration was adjusted to make the initiation rate per unit volume of oil-phase the same. Good livingness during polymerization was confirmed by the molecular weight distribution development [24,25].

A significant polymerization rate increase is observed in miniemulsion polymerization by decreasing the particle size, which leads to the conclusion that the IT model applies for the present RAFT polymerization system. The curves are the theoretical calculation results, using the IT model parameters [25]. The conclusion that the IT model applies for the dithiobenzoate-mediate styrene polymerization, rather than the SF model, agrees with the electron paramagnetic resonance (EPR) measurement results [26].

The miniemulsion polymerization is a convenient method for model discrimination. On the other hand, for the accurate estimation of kinetic parameters, bulk polymerization method would be preferable, because it is not disturbed by the existence of water phase as well as the emulsifier.

Larger polymerization rates in smaller polymer particles make it possible to conduct the *ab initio* emulsion polymerization in RAFT systems, by preventing the exit of RAFT agents from the particle. In addition, the RAFT polymerization in sufficiently small reaction loci leads to higher productivity, as in the case of conventional free-radical polymerization.

## 4. Conclusions

For conventional free-radical polymerization, the threshold particle diameter below which the polymerization rate becomes faster than the corresponding bulk polymerization was derived from the polymerization rate expression, *R*_p_ = *k*_p_[R^•^][M]. On the other hand, for RDRP, the threshold diameter below which the polymerization rate changes significantly compared with the corresponding bulk polymerization was determined from the polymerization rate expression unique to RDRP, $R_{p}=k_{p}[M]K\left[\text{RGS}\right]/\left[\text{Trap}\right]$. The obtained threshold diameters are summarized in Table 2.

For conventional free-radical polymerization, the polymerization rate increases significantly when the particle size is made smaller than the diameter given by $d_{{p,R}^{•}}^{\left(1\right)}$ in the table. Here, the superscript (1) is used to represent that the effect is caused by a high single-molecule concentration in a small particle, and the subscript R^•^ represents that the key component that causes the polymerization rate change is the polymer radical. The fact that the polymerization rate is faster for smaller particles is one of the reasons why *ab initio* emulsion polymerization is easy to conduct in the conventional free-radical polymerization.

For SRMP and ATRP, the key component to make the polymerization rate slower for smaller polymerization locus is the trapping agent, and the polymerization rate decreases significantly when the particle size is made smaller than the diameter given by $d_{p,\text{Trap}}^{\left(1\right)}$ in the table.

For RAFT, the key component to make the polymerization rate faster for smaller polymerization locus is the radical generating species (RGS), and the polymerization rate increases significantly when the particle size is made smaller than the diameter given by $d_{p,\text{RGS}}^{\left(1\right)}$ in the table. This theory can be used to discriminate two controversial models for the RAFT polymerization mechanism, *i.e.*, if the polymerization rate increases significantly in miniemulsion polymerization, the intermediate termination (IT) model applies.

## Figures and Tables

**Table 1:** Calculated single molecule concentration in a particle.

Particle Diameter *d*_p_ (nm)	Concentration (mol/L)
1000	3.18 × 10^−9^
200	3.97 × 10^−7^
100	3.18 × 10^−6^
50	2.55 × 10^−5^
25	2.04 × 10^−4^

**Table 2:** Threshold diameter below which the polymerization rate changes significantly compared with the corresponding bulk polymerization.

Type of Polymerization	Threshold Diameter	Polymerization Rate	
Conventional FRP	$d_{{p,R}^{\text{•}}}^{\left(1\right)}={\left(\frac{6\bar{n}}{\pi N_{A}{\left[R^{•}\right]}_{\text{bulk}}}\right)}^{1/3}$	Increases for *d*_p_ smaller than $d_{{p,R}^{\text{•}}}^{\left(1\right)}$	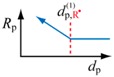
SRMP, ATRP	$d_{p,\text{Trap}}^{\left(1\right)}={\left(\frac{6}{\pi N_{A}{\left[\text{Trap}\right]}_{\text{bulk}}}\right)}^{1/3}$	Decreases for *d*_p_ smaller than $d_{p,\text{Trap}}^{\left(1\right)}$	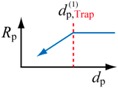
RAFT	$d_{p,\text{RGS}}^{\left(1\right)}={\left(\frac{6\bar{n}\left(1-\phi_{\text{act}}\right)}{\pi N_{A}{\left[\text{RGS}\right]}_{\text{bulk}}}\right)}^{1/3}$	Increases for *d*_p_ smaller than $d_{p,\text{RGS}}^{\left(1\right)}$	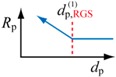

## Abbreviations

The following abbreviations are used in this manuscript:

AIBN	2,2-azobisisobutyronitrile
ATRP	Atom-Transfer Radical Polymerization
IT model	Intermediate Termination model
RAFT	Reversible-Addition-Fragmentation chain-Transfer
RDRP	Reversible-Deactivation Radical Polymerization
RGS	Radical Generating Species
SF model	Slow Fragmentation model
SRMP	Stable-Radical-Mediated Polymerization
